# Safety and Success of Extracting the Cataract in Old Age

**Published:** 1835

**Authors:** 


### III.—Safety and success of extracting the Cataract in old age.

Very lately we extracted a cataract from the eye of a woman sixty-four years old, which was followed by no more inflammation than was requisite to adhesion, although she had not been subjected to any previous preparation, either medicinal or dietetic. Last year we performed a similar operation on a man seventy-five years of age, who recovered in a favorable manner. Four or five years since, we performed the same operation on a man seventy-four years old, whose eye became inflamed for a while, but he had long been subject to ophthalmia. About the same time, we performed the same operation on a man in his eighty-sixth year, who in a week was able to see, and recovered with the least possible inflammation. From these cases we may infer, that violent and obstinate inflammation is even less likely to supervene on this operation, in advanced than middle and early life; a fact that should induce the friends of the aged, not to abandon them to the gloom of total blindnes in the evening of life.

